# A comprehensive analysis of food insecurity in the drought–prone rural areas of Tigray

**DOI:** 10.1186/s41043-024-00564-w

**Published:** 2024-05-16

**Authors:** Tewelde Gebre, Zenebe Abraha, Amanuel Zenebe, Woldegebrial Zeweld

**Affiliations:** 1 Mekelle University - Institute of Environment, Gender, and Development Studies (IEGDS), Mekelle City, Ethiopia; 2 Mekelle University-CDANR, Mekelle City, Ethiopia; 3 Mekelle University-ICS, Mekelle City, Ethiopia

**Keywords:** Food insecurity, Rural, Food availability, Access to food, Utilization, Stability

## Abstract

**Background:**

The number of globally food-insecure people is increasing since 2017. Sub-Saharan Africa has the highest proportion of severely food-insecure people in the world. Tigray region of Ethiopia is one of the food-insecure regions, which, over the past many decades has been affected by recurrent food insecurities. In the drought–prone rural areas of Tigray, many people are living under the condition of chronic hunger. Proper food security studies are vital for proper intervention mechanisms. Yet, previous food security studies have rarely addressed the four pillars of food security: availability, access, utilization, and stability. In this study, all components are duly considered to assess the food insecurity status in the drought–prone rural areas of Tigray, Ethiopia. Of the 34 rural districts in Tigray, 363 households from three drought–prone rural districts were studied.

**Results:**

Household Food Insecurity Access Scale and Food Insecurity Experience Scale were adapted to measure the food availability, access to food, and stability components of food security; and, Household Dietary Diversity Score (HDDS), Food Consumption Score (FCS), mid-upper arm circumference, and Bitot’s spot were used to analyze the food utilization aspect. Findings show that 68% of the studied community frequently ate less food than they felt they needed and 82.1% of the households have experienced hunger because of lack of food. The study rural districts were unconnected to road networks; hence, 87.9% of the elderly and 20.4% of the women and girls had no access to food markets. Regarding the food utilization, 81.5% of the studied households had poor FCS; and the average HDDS and FCS for the study areas were 2.47 and 18.9, respectively. The prevalence of Global acute malnutrition, severe acute malnutrition (SAM), and moderate acute malnutrition (MAM) for 6–59 months of age children in the study areas were 50.3, 4.2, and 46.1%, respectively. More notably, the prevalence of SAM for children from the food-insecure households was 21.2%. The prevalence of MAM for pregnant and lactating women (PLW) in the study areas was 59.5. Further, the prevalence of Bitot’s spot among 6–59 months of age children was 1.9%. On the other hand, all the rural households had anxiety about their future food demands.

**Conclusion:**

The rural households living in the studied areas were critically food-insecure. All the measurements implied that the food insecurity situation in the study areas was unacceptably worrisome and life-threatening. This calls for an instant action to avert the occurrence of famine and starvation in the drought–prone rural areas of Tigray region. Thus, interventions should primarily target the vulnerable rural people and need to be planned based on attaining food availability first rather than concurrently addressing all components of food security. Further, due emphasis should be given to diversifying livelihood strategies of the vulnerable villagers.

## Introduction

The number of people in the world who are experiencing severe hunger is on the rise from time to time. FAO [17] reported that the number of severely food-insecure world population increased from 7.5% (623.8 million people) in 2017 to 9.2% (900.1 million people) in 2022. Further, it is estimated that close to 600 million people will be confronted with chronic hunger in 2030 [18], suggesting challenges in achieving the Sustainable Development Goals target to eliminate hunger (SDG 2) by 2030. Food insecurity affects rural people disproportionately, worldwide, food insecurity affected 33.3% of rural people in 2022 compared to 26% in urban areas [18].

Sub-Saharan Africa (SSA) has the highest proportion of severely food-insecure people in the world with a total of 310.6 million people (22.5% of the total population) facing chronic hunger in 2022 [17]. Ethiopia is one of the SSA countries with a significant number of food-insecure people. Ethiopia has gained important achievements in food security in the past two decades. According to UNDP [46], poverty rate in Ethiopia declined by about 93% from 45.5% in 2000 to 23.5% in 2016. However, hunger has been a major concern in Ethiopia over the past three to four years. In 2023, an estimated 20.1 million people were facing chronic hunger across the country with 7.4 million severely undernourished children and women [52].

Tigray is one of the food-insecure regions in Ethiopia, which, over the past many decades has been affected by recurrent food insecurities [13]. During 1984/1985, Tigray experienced severe food insecurity which caused an estimated one million people deaths (Reid, 2018). Food insecurity is still the main stressful issue in the region. In June 2022, a staggering 47% of Tigray’s population was severely food insecure [51], which is the worst stage of food insecurity compared to the earlier times. According to a research cited in the Associated Press News [4], nearly 1,400 people have died because of hunger in Tigray from November 2022 to July 2023,and hunger was associated with more than 68% of deaths in Tigray making hunger the main cause of death in the region.

In times of food insecurity, it is the rural population who suffers the most. In Tigray, most rural households rely on agriculture, which mostly are dependent on erratic rainfall, to pursue their livelihoods, and because of their limited livelihood strategies rural communities in Tigray are more vulnerable to acute undernutrition [49].

According to Woldelibanos D. Head of Tigray Region Productive Safety Net Program (PSNP) (personal communication, November 19, 2019), three rural districts in Western Tigray were classified as the only food-secure rural districts of the region, and nearly ten rural districts from Eastern, Southeastern, and Southern parts of the region were classified as severely food-insecure rural districts. In 2003, there were only 16 food-insecure rural districts out of the then 34 rural districts (at present, after administrative boundary restructuring, there are 57 rural districts) in Tigray [50]. This number increased to 31 (91%) rural districts in 2019 (Woldelibanos D., personal communication, November 19, 2019).

Owing to the limited and variable rainfall patterns in the drought–prone rural areas of Tigray, which is below 600 mm of average annual rainfall [20], three rural districts of Tigray: *Irob*, *Atsbi-wenberta* and *Hintalo-wejerat* were labeled as the most food-insecure districts by the regional government as reported by the Head of Tigray Region PSNP Office.

Yet, there is no clear data regarding the food availability, access to food, utilization (nutrition) and stability in the food-insecure rural districts of Tigray region. Clear information on food insecurity situations is vital for addressing the problem sustainably. However, decision-makers and various stakeholders have been usually challenged with incomplete and contradicting information regarding food insecurity situations [16].

Food security, as indicated by FAO [15], is achieved when all the four pillars of food security: food availability, access to food, utilization and stability are concurrently fulfilled by all people at all times. Thus, food security assessment reports are expected to reflect those food security components and their levels. In this regard, a significant number of food security assessment methods have been applied by different practitioners. However, it was difficult to understand which food security component those mechanisms were measuring and why.

Proper intervention mechanisms demand proper analysis of food insecurity. Nevertheless, most of the previous researches have analyzed food security by considering only the food availability and access to food components [29]; and the utilization and stability aspects were seldom addressed when analyzing food security. Similarly, many food insecurity studies in Ethiopia used one or combined indicators of food security where the key components of food security were not fully addressed [44].

In addition, previous food security researchers used to identify the food-insecure people in a specific geographical region. For this reason, there is no sufficient understanding about the severity and general food insecurity situation within the food-insecure rural community living in drought–prone rural areas.

Therefore, this study has come up with an explicit food insecurity status in the drought–prone rural areas of Tigray region by capturing the important components of food security. The findings of this study will contribute to the intervention mechanisms to achieve food security in the drought–prone rural areas of Tigray region, Ethiopia.

## Conceptual framework

**Fig. 1:**
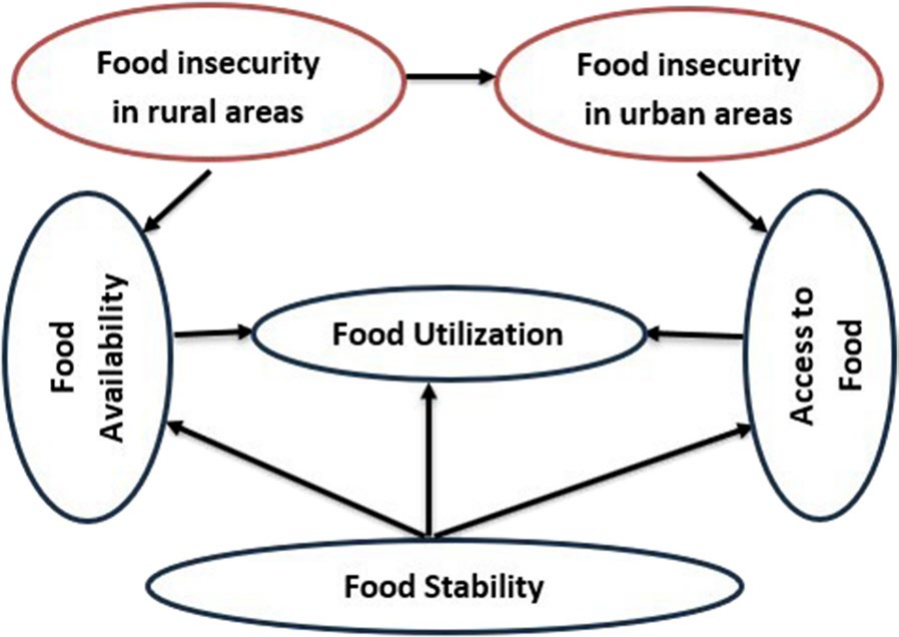
Conceptual framework of food security concept. Source: Developed by the author based on FAO [15]

As illustrated in Fig. 1, food insecurity in rural and urban areas is two distinct features as food security in rural areas is directly linked to agricultural (food) production. Because urban areas rely on non-agricultural activities, food production in rural areas has an impact on food security situation of the urban population.

According to FAO [15], food security is an aggregate measure of food availability, access to food, nutrition (utility), and stability at household or a larger level. There is a linkage in these components of food security in that food utilization can be achieved when food availability and access to food are once secured, and food stability is achieved when the other three components of food security are met.

## Methods

### Data type and sources

The study employed a cross-sectional study design based on a quantitative research approach with primary and secondary data sources. Primary data were collected through an interview-based questionnaire. The questionnaires were translated into the local language (Tigrigna and Saho) and administered through face-to-face interaction with household heads by trained enumerators (food security experts in the respective study areas); after that, a pilot survey test for possible improvement of interview questions was successfully conducted. Prior to the data gathering, participants were asked their consent to participate, and the anonymity of the respondents was ensured. On the other hand, the secondary data for the study were collected from the health offices of the respective districts.

In the survey, food consumption data was integrated with the anthropometry data for a more comprehensive and indicative food insecurity situation in the rural areas. A combination of Household Food Insecurity Access Scale (HFIAS) and Food Insecurity Experience Scale (FIES) were adapted to measure food availability, access to food and stability pillars of the food security. Additionally, Household Food Expenditure (HFE) was used to assess the economic access of the households to food. Further, Household Dietary Diversity Score (HDDS), Food Consumption Score (FCS), anthropometry measure of mid-upper arm circumference (MUAC), nutritional oedema test, and Bitot’s spot were used for measuring the utilization component of food security.

Assessment of the food security situation in rural, agriculture-based communities should be done during the period of higher food shortages [28]. The pre-harvest or agricultural lean season normally spreads between April and September. Thus, the data were gathered during May–June (2023), which falls under the most food-insecure period. Besides, based on the degree to which household food insecurity is likely to fluctuate over time, the time frame (recall period) for this study was 12 months. According to Coates [10], applications of food insecurity scales have generally used either 12 months, 6 months, or 30 days.

### Sampling techniques

This study has used both probability and non-probability sampling techniques; from the non-probability sampling techniques, a judgmental sampling method was used to select rural districts and the target population. This study is based on chronic food insecurity. Based on the purposes of the study, drought–prone rural areas with a higher proportion of food-insecure populations were selected to take sample participants for the study.

**Fig. 2:**
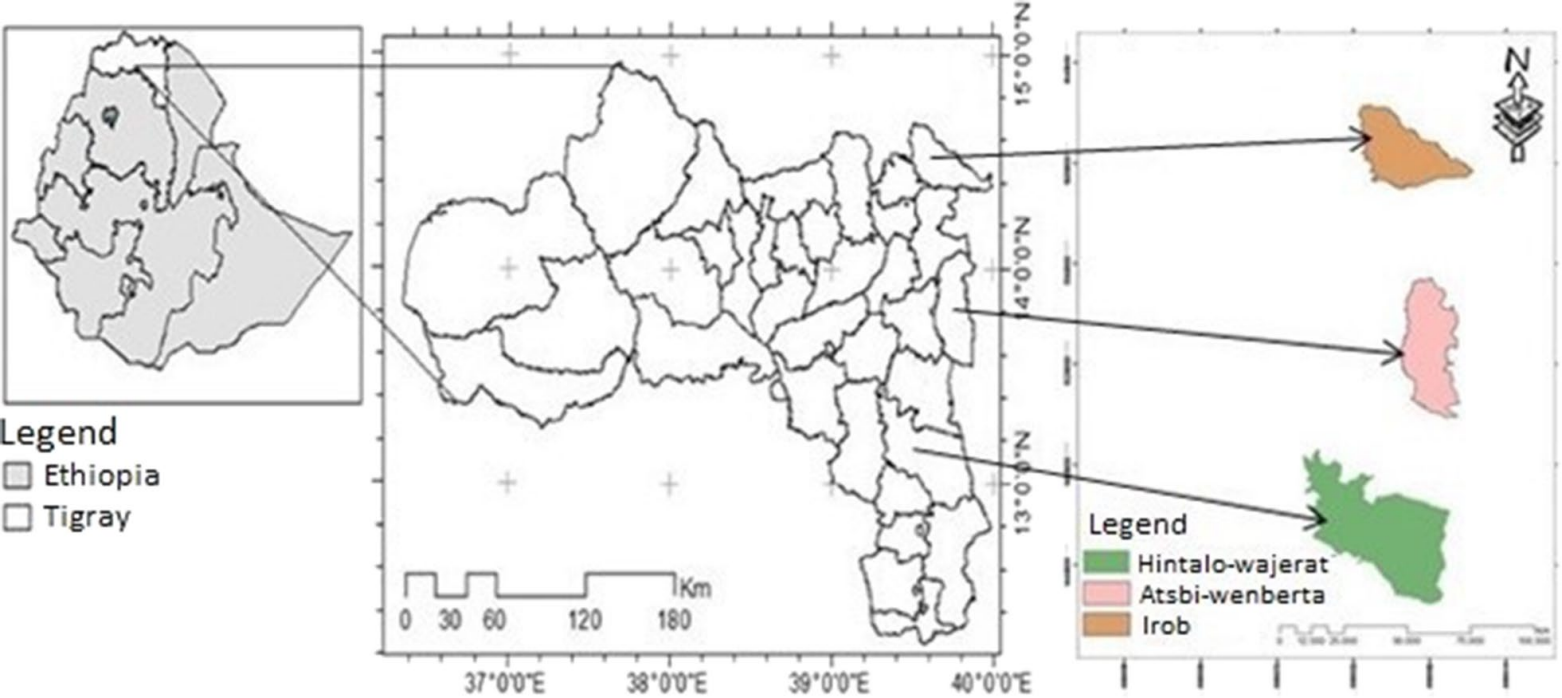
Map of the study areas

During 2021, data obtained from Tigray region office of food security shows that *Irob, Atsbi-wenberta*, and *Hintalo-wejerat* rural districts, depicted in Fig. 2, were the most drought–prone rural areas of Tigray region with a higher proportion of food-insecure population. And, two sub-districts (tabias) from each district with a higher number of food-insecure population (based on the district’s PSNP data), six sub-districts in total were selected to conduct the survey. Hence, *Alitena* and *Haraze-sebeata* from *Irob* district; *Haresaw* and *Hadinet* from *Atsbi-wenberta* district; and *Gonka* and *Seneale* from *Hintalo-wejerat* district were selected for this study.

Rural Productive Safety Net Program (PSNP) beneficiaries were the target population for this study. In these stated sub-districts, there were a total of 6528 food-insecure households being benefited by rural PSNP. This number was considered for drawing the final sample size. To draw the sample size, the following formula was adopted from the Survey Monkey [42], which is the easiest and fastest online sample size calculator for survey researches. The formula is based on margin of error and confidence level, and is convenient for a finite population.

$$n=\frac{\frac{{z}^{2}*p\left(1-p\right)}{{e}^{2}}}{1+(\frac{{z}^{2}*p\left(1-p\right)}{{e}^{2}N})}$$

where n = sample size, N = population size, e = Margin of error, and z = confidence level.

Assuming a 95% confidence level and 5% margin of error, the final sample size was found to be 363. The final sample size was distributed to each of the sub-districts proportionally as depicted in Table 1. By the end of the survey, all the filled-in questionnaires were collected.

**Table 1:** Distribution of the sample size to sampled study areas

Number	Name of the sub-district	Number of food-insecure households	Final sample size
1	Alitena	1029	57
2	Haraze-sebeata	1174	65
3	Gonka	1022	56
4	Seneale	1235	69
5	Haresaw	1080	61
6	Hadinet	988	55
Total		6528	363

Further, a simple random sampling was employed in the selection of respondent households for administering the questionnaire. This is mainly because of the availability of organized data on the number of rural PSNP beneficiaries.

### Data analysis techniques

A descriptive analysis technique (mean values, frequencies, percentiles and crosstabulation) was used to analyze the quantitative data using analysis tools in the Statistical Package for the Social Sciences (SPSS) V.27.

### Household dietary diversity score (HDDS)

HDDS is a description of the number of food groups consumed by household members in the previous 24 h. The HDDS was first developed by the Food and Nutrition Technical Assistance (FANTA) Project; as also indicated by Swindale and Bilinsky [43], it is a 12-scale level and a proxy measure of access to food by households.

$$HDDS \, = \, Sum \, \left( {A \, + \, B \, + \, C \, + \, D \, + \, E \, + \, F \, + \, G \, + \, H \, + \, I \, + \, J \, + \, K \, + \, L} \right)$$

where A to L are the 12 individual food groups representing cereals, root and tubers, vegetables, fruit, meat, egg, fish, legumes, milk, oil and fats, sweets, and spices.

Based on Swindale and Bilinsky [43], the average household dietary diversity score for the population of study can be calculated as follows:

$${\text{Sum}}\left( {{\text{HDDS}}} \right){\text{/Total}}\;{\text{number}}\;{\text{of}}\;{\text{households}}\;{\text{surveyed}}$$

### Food consumption score (FCS)

FCS is an index developed by WFP that aggregates household-level data on the diversity and frequency of food groups consumed over the last seven days [25]. FCS is calculated as:

- Grouping food items in the specified food groups;
- Summing all the consumption frequencies of food items within the same group;
- Multiplying the value of each food group by its weight; and
- Summing the weighted food group scores

Based on World Food Program’s (WFP) recommended cut-offs, FCS results of 0–21 are labeled as poor; 21.5–35 as borderline; and FCS above 35 as acceptable [25].

### Analyzing malnutrition

Based on WHO [53], Moderate Acute Malnutrition (MAM) is identified for children 6–59 months, using the standard interval measure of MUAC < 125 mm and > 115 mm).

Severe Acute Malnutrition (SAM) is identified for children 6–59 months, using the standard interval measure of MUAC < 115 mm or the presence of bilateral pitting oedema.

Global Acute Malnutrition (GAM) is the presence of both MAM and SAM of a population. A GAM value of more than 15% indicates an emergency. According to WHO [53], the thresholds for GAM are:

-  < 5%, acceptable
- 5–9.9%, poor
- 10–14.9%, serious
-  > 15%, critical

Nutritional oedema is an independent indicator of SAM in children 6–59 months of age [38]. Nutritional oedema for children 6–59 months is identified by thumb pressure on the top side of both feet gently for three seconds. The child has oedema if the indent stays after lifting the thumb for some time or more and is considered as SAM [38].

Bitot's spot is a vitamin A deficiency and is identified when a slightly elevated, white foamy lesion can usually be seen as the temporal part of the bulbar conjunctiva near the limbus at the left or right positions [30]. The presence of Bitot’s spots on children’s eyes was observed with the support of a magnifier and flashlight.

## Results

### Demographic and socio-economic characteristics of the households

Analysis of demographic and socio-economic characteristics of household heads has a pivotal importance in food security researches. A recent food security research in Gondar area shows that age and sex of household head, income, family size, asset ownership, on-farm and off-farm activities, and farmland size were significant determinants of food security at the household level [7].

**Fig. 3:**
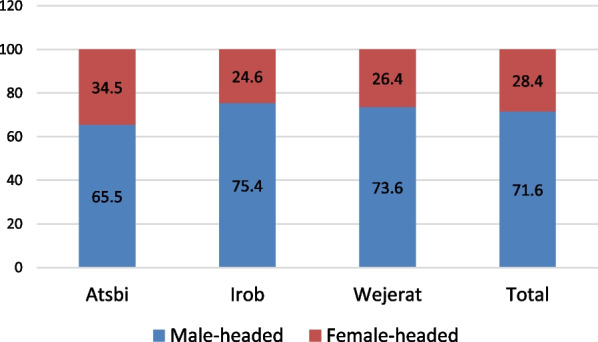
Sex of the household head

In Fig. 3, it is shown that 71.6% of the interviewed households had male heads. According to Negesse et al. [36] female-headed households (FHH) had a double increased possibility of being food-insecure when compared to male-headed households (MHH) in Ethiopia.

The average age of the household head was 46.6 years of age, as shown in Table 2. Nearly 47% of the household heads were illiterate; and, only 7% of the household heads had reached or completed grade 10.

**Table 2:** Demographic and socio-economic characteristics of households

No.	Households’ characteristics	Min	Max	Mean	Std. D
1	Age of the household head	20	80	46.60	12.48
2	Formal schooling year of the household head	0	15	3.17	3.68
3	Total number of household members (Family size)	2	11	5.40	2.12
4	Total number of female household members	1	7	2.70	1.23
5	Total number of male household members	0	9	2.75	1.75
6	Total number of under five years age children	0	3	0.92	0.81
7	Number of economically active household members	0	4	1.67	0.79
8	Average amount of annual income in ETB	1,500	16,000	5,830.6	3777.41

The average family size of households in the study areas was 5.4. This is higher when compared to the national average of 4.6 as per the 2016 demographic and health survey of Ethiopia [11]. Food insecurity among large family households in Jimma was 3.74 times higher than that of smaller family-size households [5].

The average number of economically active members per household in the study areas was 1.67. For that reason, the dependency ratio in the study areas was found to be 1.85, meaning that 100 economically independent persons supported nearly 185 persons in addition to themselves. This is much higher than the Ethiopian dependency ratio of 0.74% in 2022 as reported by Trading Economics [45].

In the study areas, about 23.1% of the households had no own farmland. In line with this, the average livestock ownership was found below one for cattle, donkeys/horses, and camels, as shown in Table 3. And, the average household ownership rate for sheep/goat and chicken was 1.6 and 2.78, respectively.

**Table 3:** Livestock and land ownership

No	Asset ownership	Minimum	Maximum	Mean	Std. D
1	Total number of cattle	0	3	0.91	0.95
2	Total number of donkeys or horses	0	2	0.32	0.49
3	Total number of camels	0	1	0.02	0.15
4	Total number of sheep and/or goats	0	10	1.6	2.06
5	Total number of chickens	0	10	2.78	2.73
6	Farmland size in hectares	0	1	0.21	0.73

**Fig. 4:**
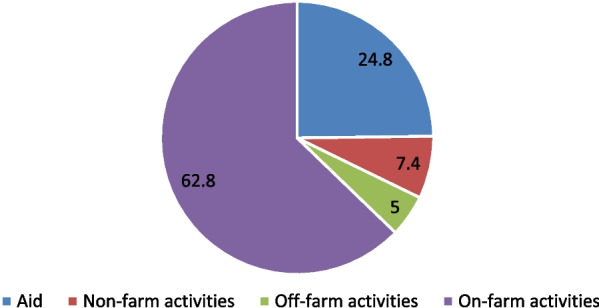
Main income source of households

Rural people rely mainly on agricultural activities to earn income. As indicated in Fig. 4, on-farm activities were the main source of income for the households followed by humanitarian aid. Yet, 98.8% of the interviewed smallholder households were dependent on rain-fed agriculture (data not shown). Besides, as revealed in Table 3, the average farmland size of the households was 0.21 hectares.

**Fig. 5:**
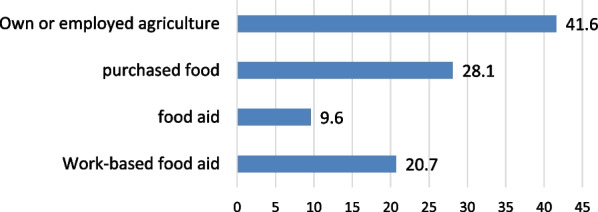
Main food source of the studied households

The average annual income of the households under the study was ETB 5,830 (USD 104.13, as of December 28, 2023).^1^ This is far below the global low-income threshold of USD 1,045 and less [56]. Concerning the source of food, agriculture was the main source of food for a majority (41.6%) of the households and nearly 28% of the households get their food from the market, as shown in Fig. 5.

**Fig. 6:**
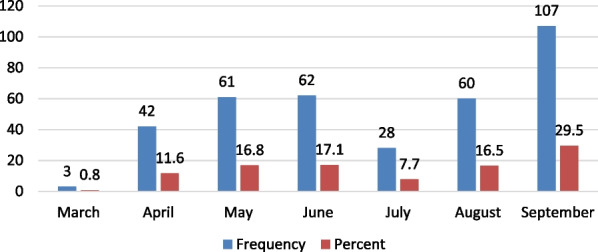
Month of chronic hunger

According to 29.5% of the surveyed households, September was a month of chronic hunger followed by June, May, and August, as depicted in Fig. 6.

### Analysis of food insecurity (2023)

Based on the 1996 World Food Summit, all the four dimensions of food security, that is, food availability, access to food, utilization (nutrition) and food stability must be fulfilled simultaneously by all people, at all times for the realization of food security objectives (FAO, 2008). To meet these requisites for food security, food insecurity in the drought–prone rural areas of Tigray is analyzed by giving due consideration to the four pillars of food security, as discussed below.

### Food availability

In the drought–prone rural areas of Tigray, many rural villagers have been suffering from chronic food shortages. Table 4, indicated that 66.7% of the households mostly cut the size of their meal and/or have been eating less than they felt they should. In addition, more than 90% of the households reported that there were times when there was no food at all to eat in their house and more than 80% of the households had felt hunger because there was no food at the household.

**Table 4:** Household’s response regarding their food availability status over the last year

No	Questions	Yes	No	How often did this happen?		
				Everyday	Sometimes	Rarely
1	Did you or any household member ever eat less than you felt you should?	100	0.0	68.0	7.7	24.3
2	Did you or any household member ever cut the size of the Household meals?	100	0.0	66.7	15.1	18.2
3	Was there ever no food to eat of any kind in your household because of a lack of resources to get food?	92.3	7.7	8.1	20.3	71.6
4	Were you or any household member ever hungry?	82.1	17.9	6.3	14.9	78.8
5	Were you or any household member not able to eat the kinds of foods you preferred because of a lack of resources?	98.9	1.1	92.0	5.8	2.2
6	Did you or any household member have to eat a limited variety of foods due to a lack of resources?	100	0.0	81.0	12.6	5.4

A similar food security study in Tigray by Weldegiargis et al. [48] confirmed that nearly 75% of interviewed households were eating smaller meals or went a whole day without eating any food.

### Access to food

All the market centers of the study areas, i.e., *Atsbi*, *Dowhan* and *Bahre-tseba* were accessible from the main road through solely a low-quality earthen road of 25 km, 80 km, and 34 km long, respectively. Nevertheless, all the interviewed households were unconnected to any road transport; and they had to walk for an average of one hour and forty-three minutes to get to the nearest food market. In some remote rural areas of *Irob* district, the villagers had to walk for nearly four hours. For this reason, only 12.1% of the elderly rural population of the study areas can access the food market. And, a significant (20.4%) number of households in the study areas, in which women and girls take on such tasks, had no access to the food market, as shown in Table 5.

**Table 5:** Household’s response regarding their access to food over the last year

No	Questions	Yes	No
1	Do the elders have access to food market?	44 (12.1%)	319 (87.9%)
2	Do the women and girls have access to food market?	289 (79.6%)	74 (20.4%)
3	Was the money you spent enough to purchase your required food?	0 (0%)	363 (100%)
4	Were you or any household member hungry because there was no money to buy food?	359 (98.9%)	4 (1.1%)

The other key determinant of food security is economic or financial access to food. Globally, nearly 3.1 billion people cannot afford a healthy diet [25]. According to the Global Food Security Index [23], Ethiopia was ranked 108th out of 113 countries with least food affordability of 32.9%. In 2022, 85% of the rural households in Tigray were unable to afford food prices because of a lack of money to purchase food [51]. In addition, prices of cereals and pulses in Tigray have been remarkably high when compared to prices in Dessie,and, the prices of teff, maize, sorghum, wheat, rice, and fava beans have risen steeply in Tigray markets [51].

In this study, 28.1% of households relied on purchased food, and an average of ETB 586.7 was incurred monthly by the households to purchase food. In this regard, all the interviewed households replied that the money they spent on food was not enough to buy the required food. In line with this, 98.9% of the studied households claimed that they have experienced hunger because there was no money to purchase food, as shown in Table 4.

### Food utilization

One way of assessing food utilization at the household level is by looking at dietary diversity. 94.2% of the households reported that they have been eating two or fewer food varieties in a day. Besides, 92% of the households claimed that they were unable to eat the kinds of food they preferred, and 81% of the households had to eat a limited variety of foods because of lack of resources. In 2016, 23% of rural households in Tigray had low food diversity [40].

**Fig. 7:**
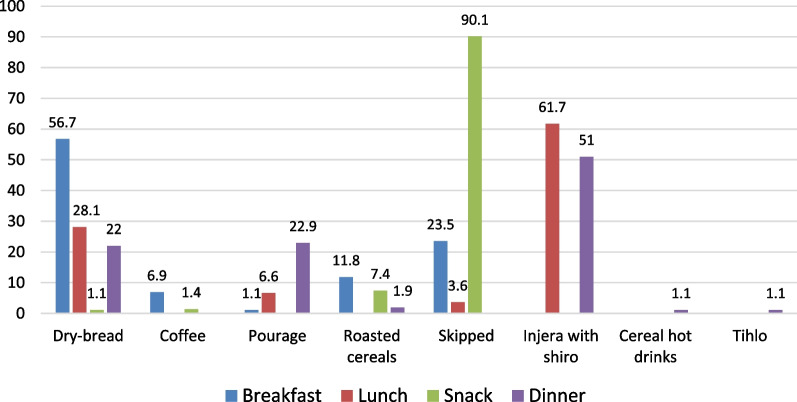
Consumption pattern of staple foods in the study areas

As shown in Fig. 7, dry-bread made of wheat flour, *Injera*^2^ with *Shiro*,^3^ porridge made of various cereals and roasted cereals are the food staples of the study areas. This shows that almost all the households in the study areas consume two food groups: cereals and legumes, which indicates that their dietary diversity is below the minimum dietary diversity required level of four to five food groups [55].

*Injera* is a traditional staple food in Ethiopia and Tigray in particular. According to Neela and Fanta [35], different studies have been conducted on the composite flour development of *Injera* for its better nutritional and sensory quality; and few studies reported the fermentation process had contributed to a reduction of nutritional quality and mineral availability in injera.

**Fig. 8:**
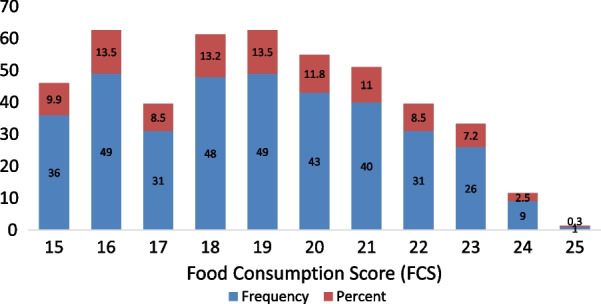
Food Consumption Score of studied households

In this study, the average Food Consumption Score (FCS) was found to be 18.9 at the time of the survey. According to IDEP [25], household's FCS below 21 is categorized as poor. Figure 8 shows that 296 (81.5%) of the households had a poor FCS of 21 and below, and the rest had a borderline FCS of 21.5–25. According to food insecurity research findings based on FCS, more than half of the sampled households in Ethiopia were food-insecure [2, 19, 41].

The Ethiopian socio-economic survey data reported by Jateno et al. [26] revealed that cereals and pulses were the most dominant food groups consumed by 96.4 and 82% of the households in Ethiopia, respectively. The same source reported that food commodities like meat and fruits were the least consumed food groups by households in Ethiopia.

**Fig. 9:**
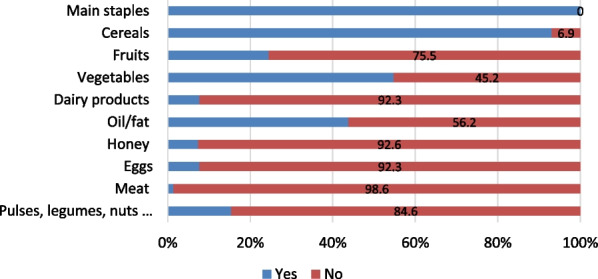
Weekly food diversity consumption

Figure 9 indicates more than 90% of the households did not consume important diets like meat, egg, dairy products, and honey for seven days before the survey. As a result, the average Household Dietary Diversity Score (HDDS) for this study was found to be 2.47. According to Kennedy et al. [28], HDDS values less than five are labeled as low or poor food diversity. In rural Ethiopia, the average HDDS value for the year 2022 was 5.52 [27]. Similarly, the mean dietary diversity of pregnant and lactating women in Ethiopia in 2020 was 3.99,and 65.7% of the pregnant women were found to have poor food diversity [8].

**Fig. 10:**
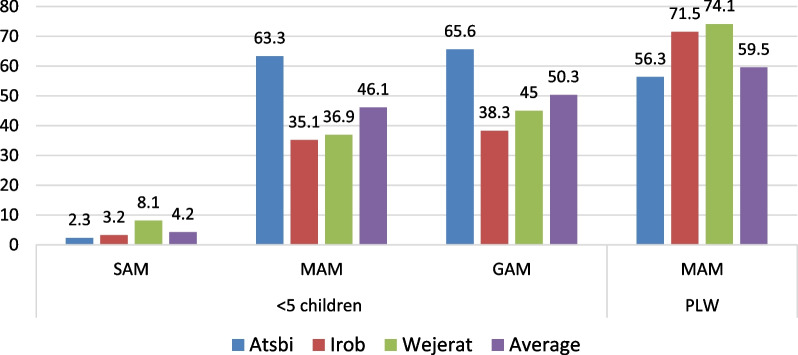
Prevalence of acute malnutrition among 6–59 months of age children and PLW based on MUAC

In the selected study areas, data obtained from the health office of the respective districts revealed that, of the screened 11,321 6–59 months of age children, more than half of them (5698 children) were affected by acute malnutrition during December 2023; of which 4.2% and 46.1% of them were affected by SAM and MAM, respectively. On the other hand, of the screened 10,446 pregnant and lactating women (PLW), 6213 (59.5%) were affected by acute malnutrition during December 2023, as illustrated in Fig. 10.

In this study, 265 children with 6–59 months of age from food-insecure rural households were surveyed to test the level of malnutrition based on MUAC and oedema; of which 159 (60%) of them were found with acute malnutrition and 56 (21.2%) of the children were affected by SAM. In line with this, Ghimire et al. [22] reported that Children from severely food-insecure households in Ethiopia were four times more likely to be affected by SAM.

Vitamin A Deficiency (VAD) is among the health problems of developing countries. In Ethiopia, VAD was recognized as a public health problem in the 1970s; and studies showed a higher prevalence of VAD in Ethiopia which is higher than WHO standards [58]. Bitot’s spot was used in this study to assess VAD and its prevalence was 1.9%. According to Yisak et al. [58], The prevalence of Bitot’s spot in Ethiopia in 2019 was 0.8%. The value of prevalence of Bitot's Spot more than 0.5% is used as the WHO standard point to declare that VAD is a public health issue [54].

### Food stability

Because of limited food availability, securing stable food access is unthinkable in food-insecure rural households. As shown in Table 6, 94.8% of the rural households in the study areas reported during the survey that they had stored food. However, 76.7% of these households had stored food that could last only for days; and the rest of them had stored food that could last for weeks. Thus, all the households were uncertain about their food consumption after a month. As a result, all the households had anxiety about having sufficient future food to feed themselves and the children, mothers, and elders in particular. Besides, 97.8% of the households were worried about getting a variety of foods for the future. Likewise, a food insecurity study by Weldegiargis et al. [48] reported that 75% of the households in Tigray had experienced anxiety and uncertainty about food supply in 2021.

**Table 6:** Household’s response regarding food stability

No	Questions	Yes	No
1	Do you have any stored food for future use?	344 (94.8%)	19 (5.2%)
2	Did you worry that your household would not have enough food?	363 (100%)	0 (0%)
3	Did you worry that your household would not have enough money to purchase food?	363 (100%)	0 (0%)
4	Did you worry that any of the children, women, or the elders would not have enough food?	363 (100%)	0 (0%)
5	Did you worry that your household would not have a variety of foods (like meat, egg, milk & milk products …)?	355 (97.8%)	8 (2.2%)

## Discussion

Rural people are chief food producers; In many societies, rural people are the primary vendors at food markets. By selling their food products, they contribute to local economies and ensure food accessibility to urban areas. Paradoxically, rural people who produce most of the food supply are more food-insecure in many developing nations when compared to urban people. According to Ahmadi et al. [1], 80% of the rural people in Iran suffered from food insecurity in 2020.

In Ethiopia, people living in drought–prone rural areas are at greater risk of food insecurity. According to Asrat and Anteneh [6], about 13% of Ethiopian people live in drought–prone rural areas and many of these households suffer from chronic undernourishment and food insecurity. Food security research in the arid and semi-arid areas of Southern Ethiopia by Eshetu and Guye [14] indicated that the prevalence of food insecurity in these areas was 68%, and the average vulnerability to food insecurity was 73%. Similarly, about 64% of the sampled households living in North-eastern rift valley of Ethiopia, which is the other drought–prone area of Ethiopia, were found to be food insecure [21].

In rural areas, having physical, economic, and social access to food is crucial for livelihoods and is among the basic requirements for achieving food security. Elderly and vulnerable population in rural communities often requires physical access to the nearest urban and peri-urban areas for marketing purposes. According to Nakamura et al. [34], rural road development in Ethiopia was associated with a significant increase in household welfare. Yet, gaining physical access to the food market is the main challenge for rural communities.

Despite the promising progress in road infrastructure, a greater proportion (about 40%) of the national populations living in Bangladesh, Nepal, Kenya and Uganda, Ethiopia, Mozambique, Tanzania, and Zambia are suffering from remoteness [12]. According to Nagesso et al. [33], only 10% of the rural population in Ethiopia lives within two kilometers of an all-weather road. In rural Ethiopia, the average distance of rural communities to the nearest market was estimated to be 7.82 km [37].

Similar research in Dedo district of Oromia region shows that more than 65.9% of the villagers had to travel about 4 to 5 km distance from their home to the next main road and 21.6% of respondents revealed that the distance from the nearest main road to market was 6 to 7 km [57]. In 2022, more than 75% of the households in Tigray reported that they do not have physical access to markets [51].

Food utilization (nutrition), the other key pillar of food security, is essential for healthy development. Better nutrition is related to improved infant, child and maternal health, stronger immune systems, safer pregnancy and childbirth, and lower risk of non-communicable diseases. The main topics in food research discourses lately have been food utilization, nutrition, and safety.

Balanced nutrition is particularly vital during childhood. The physical and mental development of infants and children is dependent on adequate nutrition [39]. Nevertheless, based on UNICEF, WHO and World Bank [47] joint child malnutrition estimate of 2021, 149.2 million children in the world under the age of 5 were stunted and 45.4 million were wasted (low weight-for-height). The same report shows that the number of stunting children is declining in all parts of the world except in Africa.

Acute malnutrition is a persistent public health issue affecting the world population disproportionately. In 2016, there were 32.8 million children worldwide affected by Moderately Affected Malnutrition (MAM), of which about 97% of them were living in underdeveloped countries; and there were 18.7 million children worldwide affected by Severe Acute Malnutrition (SAM), of which about 99% of them were living in underdeveloped countries [9]. Similarly, there were nearly 17 million under-five children worldwide affected by SAM in 2018,of which 75% of them were living in low-income countries [47].

In Ethiopia, the prevalence of MAM and SAM in under-five children in 2021 was 18 and 8%, respectively [3]. Similar research finding by Ghimire et al. [22] shows that the prevalence of SAM in under-five children in Ethiopia in 2019 was 5.8%. According to WHO [53] document, a Global Acute Malnutrition (GAM) value of more than 15% is categorized as a critical severity of malnutrition and indicates an emergency.

In Tigray, an acute malnutrition assessment based on MUAC and oedema by WFP [51] reported that the prevalence GAM, MAM, and SAM in 2021 among under-five children was 29.4, 23.6 and 5.8%, respectively,and 53.6% of lactating women and 59.6% of pregnant women were affected by acute malnutrition. Similarly, a rapid nutritional assessment by Mulugeta and Gebregziabher [32] showed that 28% of children aged 6–59 months had GAM, and 6% had SAM.

Food stability is the other significant component of food security. According to food security concept of FAO (2008), food stability occurs when the other three components of food security, that is, food availability, access to food and utilization are secured by all households or individuals at all times even in times of bad events. Similarly, Helland and Sørbø [24] explained food stability as the sustainability in the availability of food, access to food, or ability to purchase food and sufficient nutrition. In the Sustainable Development Goals of 2030, the significance of food stability is highlighted: “SDG 1: End of poverty” and “SDG 2: Zero hunger” describe the food stability component of food security.

Apart from achieving food security at all levels, achievement of food security also requires a time dimension. According to Maxwell and Smith [31], food insecurity can be categorized as “chronic”: a continuous failure to get food, or “transitory”: a temporary failure to get food. Consistent handling of food security thus requires integrating time and spatial dimensions.

For the reason that food stability is related to the other three food security pillars, any activity in rural areas oriented to achieve sustainable food availability, access to food and utilization will contribute to the attainment of food stability in particular and food security in general.

## Strengths and limitations of the study

This study has come up with an explicit food insecurity status in the drought–prone rural areas of Tigray region by capturing the important components of food security: availability, access, utilization, and stability. The findings of this study will help to guide the intervention mechanisms to achieve food security in the drought–prone rural areas of Tigray region, Ethiopia.

In this study, all the anthropometric measurements were not taken to measure child malnutrition; and the calorie intake of the food-insecure households was not measured. Hence, analyzing the stunting (low height-for-age) and wasting (reduced height-for-weight) for under five-years age children and measuring the calorie intake of these food-insecure rural households is left for future researchers interested in conducting similar food security studies. More studies on specific nutritional indicators and household calorie intakes will significantly contribute to a more comprehensive understanding of food security.

## Conclusion

The food security situation in the drought–prone rural areas of Tigray region is very life-threatening and needs an instant action. In all measurements, the households living in the studied areas were critically food-insecure. Most of the food-insecure households rely on their farm activities to meet their food requirement where the farmland size is too small to support their larger family size. This signals that the high food insecurity problem encountered in the area shall not be left unattended and requires diligent actions.

The food availability and hunger levels of the food-insecure households were unacceptably worrisome. Because of limited food supplies, almost all the study rural communities had to eat a lesser amount of food which might be the cause of famine and starvation. It is even more challenging for these rural communities to get access to food because of their limited physical, social and economic capacities. In times of food shortages, the study communities had to walk for longer and more challenging distances to purchase food with the inadequate money in hand.

Both the FCS and HDDS results show a limited variety of food consumption below the standards. For this reason, 6–59 months of age children living in the food-insecure rural areas are at higher risks than any other age group. The SAM, MAM and GAM results were unacceptably high, which are greater than the WHO cut-off points for an emergency. Besides, because of the higher prevalence of Bitot’s spot above the WHO standard, VAD in the food-insecure study areas is a public health concern. This calls for immediate attention and implies that much more should be improved to curb the serious food insecurity prevailing in the study areas.

More importantly, the study communities had very limited or no food supplies for their future use. In this situation, the food insecurity status of the communities living in these drought–prone rural areas will get worse and might cost us lives. Thus, an integrated life-saving task is required provisionally and diversifying livelihood sources of the food-insecure rural communities is a “must-to-do” activity to sustainably achieve food security. In the due course, priority should be given to attaining the food availability and access components of food security.

This study implies that long-term food security attainment plans are much required to guide the intervention mechanisms to be undertaken by the government of all levels, international communities, and humanitarian agencies. In the meantime, elaborated short-term strategies are needed to address the child malnutrition and households’ food shortage in the drought–prone rural areas of Tigray.

## Data Availability

We declare that data and materials will be made available on reasonable request.

## Footnotes

1 One USD = ETB 56.148.

2 a fermented flatbread traditionally made of unique or mixed cereals of teff, wheat, barley, sorghum, maize etc. flours.

3 ground chickpeas or broad bean stew.
